# Functionalization of breast implants by cyclodextrin *in-situ* polymerization: a local drug delivery system for augmentation mammaplasty

**DOI:** 10.3389/fbioe.2023.1254299

**Published:** 2023-09-19

**Authors:** Karen Escobar, Ignacio Carrera, Nelson Naveas, Ruth Pulido, Miguel Manso, João Paulo de Oliveira Guarnieri, Marcelo Lancellotti, Monica A. Cotta, Yendry Regina Corrales-Ureña, Klaus Rischka, Jacobo Hernandez-Montelongo

**Affiliations:** ^1^ Department of Mathematical and Physical Sciences, UC Temuco, Temuco, Chile; ^2^ Department of Applied Physics, Centre for Micro Analysis of Materials and Nicolás Cabrera Institute of Materials Science, Autonomous University of Madrid, Madrid, Spain; ^3^ Departamento de Ingeniería Química y Procesos de Minerales, Universidad de Antofagasta, Antofagasta, Chile; ^4^ Departamento de Química, Universidad de Antofagasta, Antofagasta, Chile; ^5^ Faculty of Pharmaceutical Sciences, State University of Campinas, Campinas, Brazil; ^6^ Institute of Physics Gleb Wataghin, State University of Campinas, Campinas, Brazil; ^7^ National Laboratory of Nanotechnology, San José, Costa Rica; ^8^ Fraunhofer Institute for Manufacturing Technology and Advanced Materials, Bremen, Germany; ^9^ Department of Translational Bioengineering, University of Guadalajara, Guadalajara, Mexico

**Keywords:** breast implants, in-situ polymerization, drug delivery, cyclodextrin, silicone

## Abstract

Mammaplasty is a widely performed surgical procedure worldwide, utilized for breast reconstruction, in the context of breast cancer treatment, and aesthetic purposes. To enhance post-operative outcomes and reduce risks (hematoma with required evacuation, capsular contracture, implant-associated infection and others), the controlled release of medicaments can be achieved using drug delivery systems based on cyclodextrins (CDs). In this study, our objective was to functionalize commercially available silicone breast implants with smooth and textured surfaces through *in-situ* polymerization of two CDs: β-CD/citric acid and 2-hydroxypropyl-β-CD/citric acid. This functionalization serves as a local drug delivery system for the controlled release of therapeutic molecules that potentially can be a preventive treatment for post-operative complications in mammaplasty interventions. Initially, we evaluated the pre-treatment of sample surfaces with O_2_ plasma, followed by chitosan grafting. Subsequently, *in-situ* polymerization using both types of CDs was performed on implants. The results demonstrated that the proposed pre-treatment significantly increased the polymerization yield. The functionalized samples were characterized using microscopic and physicochemical techniques. To evaluate the efficacy of the proposed system for controlled drug delivery in augmentation mammaplasty, three different molecules were utilized: pirfenidone (PFD) for capsular contracture prevention, Rose Bengal (RB) as anticancer agent, and KR-12 peptide (KR-12) to prevent bacterial infection. The release kinetics of PFD, RB, and KR-12 were analyzed using the Korsmeyer-Peppas and monolithic solution mathematical models to identify the respective delivery mechanisms. The antibacterial effect of KR-12 was assessed against *Staphylococcus epidermidis* and *Pseudomonas aeruginosa*, revealing that the antibacterial rate of functionalized samples loaded with KR-12 was dependent on the diffusion coefficients. Finally, due to the immunomodulatory properties of KR-12 peptide on epithelial cells, this type of cells was employed to investigate the cytotoxicity of the functionalized samples. These assays confirmed the superior properties of functionalized samples compared to unprotected implants.

## 1 Introduction

Breast cancer is the most diagnosed cancer and the one with the highest death rate (Arnold et al., 2022). According to the Global Cancer Observatory, in 2020, over 2 million new cases around the world of breast cancer were diagnosed, resulting in over 30% deaths within the first year (Global Cancer Observatory, 2020). Most women diagnosed with this type of cancer undergo a surgical treatment called mastectomy, which consists of the partial or total removal of one or both breasts. Consequently, the result of this surgery can compromise them physically, emotionally, and socially. For this reason, reconstruction options such as augmentation mammaplasty are used, which helps to reduce the sequelae of mastectomy, allowing to improve satisfaction, self-esteem, body image, and in general, the quality of life of patients (Atisha et al., 2008).

Augmentation mammaplasty is a cosmetic surgery that consists of increasing or restoring the size of the breasts using silicone implants, which can be filled with a silicone gel or a sterile, aqueous saline solution. It is one of the most common surgical procedures performed today, since it is performed both for reconstructive surgery in post-breast cancer treatment and for pure cosmetic reasons (Abdul-Al et al., 2020). According to the International Society of Aesthetic Plastic Surgery (ISAPS), breast augmentation continues to be one of the most popular surgery worldwide, with over 1.5 million interventions that represented 13.1% of all surgical procedures in 2021 (The Int ernational Society of Aesthetic Plastic Surgery, 2021). Although reconstruction offers considerable benefits for the recovery of patients with breast cancer, various complications have been described for these procedures, such as bruising, rupture or emptying of implants, capsular contracture, and bacterial infections (Pittet et al., 2005; Maxwell and Gabriel, 2012). Since the introduction of silicone implants, new designs have been made to increase the biocompatibility of the implants and reduce postoperative problems (Holzapfel et al., 2013). Five different generations of implants have been developed so far (Bergmann et al., 2014). However, these still experience complications (Teck Lim et al., 2013); therefore, to minimize postoperative complications, it is necessary to modify the implants available on the market in order to improve their properties and biocompatibility.

There are different types of breast implants in terms of filling, size, shape, and surface topography. In the case of surface topography, implants with smooth and textured surfaces are commercialized. The smooth surface allows implants to move within the chest, simulating the breast’s natural movement. However, this displacement capacity prevents the implants from adopting a stable position, causing a lateral deviation of these and their settlement in the lower part of the pocket; can stretch lower pole over time (Calobrace et al., 2018). Furthermore, the smooth surface has been associated with high rates of fibrosis. However, these implants are still being used because they provide a perfect circular shape of the breasts, making them visually more natural (Shin et al., 2018). On the other hand, textured implants have higher control of implant movement through an adhesive effect. The roughness of the surface induces the formation of a capsule of collagen tissue around the implant that facilitates its fixation. These implants are intended for reducing the incidence of capsular contracture, and although there are studies that support the effectiveness of these implants, there is still controversy regarding their benefits (Adams, 2009; Chang and Hammond, 2018).

In the context of a patient’s quality of life, a viable alternative to modify the surface of an implant is the use of cyclodextrins (CDs). These molecules are a family of cyclic oligosaccharides with a hydrophilic outer surface (C-OH groups) and a hydrophobic apolar cavity (C-O-C and C-H bonds) (Mura, 2015). They do not present toxicity, have good solubility, are easily modifiable, and have a high biological availability (Zhang et al., 2019). In addition, they are available to generate controlled release systems due to their characteristic cavity capable of forming reversible complexes with various drugs (Sherje et al., 2017). Additionally, CDs can be crosslinked with bi- or multifunctional agents to form polymers (Concheiro and Alvarez-Lorenzo, 2013), which consist of a three-dimensional network suitable for drug delivery applications (Hernández-Montelongo et al., 2014). These systems substantially increase the stability of drugs and decrease side effects in the body, resulting in a specific, safe, and effective medication to reduce the development of possible postoperative complications. For these reasons, CD-based polymers have been used to functionalize different biomedical materials for drug release applications, such as mesh implant for parietal reinforcement, titanium hip prostheses (Vermet et al., 2017), porous bioceramics (Taha et al., 2014), paper points for periodontal pockets treatment (Chai et al., 2014), and others.

Currently, there are no reports in the literature regarding the application of cyclodextrin biopolymers as coatings on silicone breast implants for drug delivery. However, there are some reports about other type of polymers that have been used, for example, Kim et al. (2019) (Kim et al., 2019) that used patterned coating dots of poly (lactic-co-glycolic acid) (PLGA) to release tranilast for the fibrosis suppression. In the same sense, Kang et al. (2019) (Kang et al., 2020) heat-induced polymerization of 2-methacryloyloxyethyl phosphorylcholine on silicone breast implants for the inhibition of capsular formation and inflammation. Moreover, Gosau et al. (2013) (Gosau et al., 2013) coated silicone material used for breast augmentation with different copper concentrations to study the anti-adherence and bactericidal effects on *S. epidermidis*.

Based on the above, this work focuses on a novel functionalization tool for commercial silicone breast implants, with smooth and textured surfaces, by β-cyclodextrin/citric acid and 2-hydroxypropyl-β-cyclodextrin/citric acid *in situ* polymerization as a preventive treatment for mammaplasty postoperative risks. Firstly, a pretreatment of oxygen plasma oxidation with subsequent chitosan grafting was evaluated. Then, polymerization stability on samples was studied and physicochemically analyzed. Three molecules that could be useful in augmentation mammaplasty surgery, were used as model to test the obtained samples as controlled local drug delivery system for mammaplasty: pirfenidone (PFD) proposed for capsular contracture prevention (Veras-Castillo et al., 2013; Guimier et al., 2022), Rose Bengal (RB) that possesses cytotoxic properties in cancer cells (Loya-Castro et al., 2018; Demar et al., 2021), and KR-12 peptide, which is antimicrobial and has antibiofilm properties to prevent bacterial infections (M et al., 2019; Ajish et al., 2022; Liu et al., 2019). Previous works have reported the successful complexation CDs and these molecules: PFD with β-cyclodextrin (Ji et al., 2016; Panigrahi et al., 2023), RB with hydroxypropyl-cyclodextrins (Fini et al., 2007) and β-cyclodextrin (Khushbu and Jindal, 2022), and KR-12 with β-cyclodextrin (Consuegra et al., 2013). The kinetic release mechanism of the used model molecules was studied by the Korsmeyer-Peppas, and monolithic solution mathematical models. Furthermore, the antibacterial effect of KR-12 was tested against *Staphylococcus epidermidis* and *Pseudomonas aeruginosa* bacteria, and the cytotoxicity of functionalized samples was studied using epithelial cells. It is of significance to emphasize that the KR-12 peptide constitutes the most concise antimicrobial motif within the human cathelicidin LL37 (Wang, 2008; Ramos et al., 2011). Remarkably, it not only demonstrates antibacterial activity but also possesses immunomodulatory properties on epithelial cells (Ramos et al., 2011; Lei et al., 2023). The conducted assays have corroborated the heightened attributes of functionalized samples when compared to unprotected implants.

## 2 Materials and methods

### 2.1 Materials

Commercial silicone implants with a smooth and textured surface (300 cc and 375 cc, respectively) were purchased from Mentor^®^ brand (United States). Chitosan (CHI, 75%–85% deacetylated, low molar mass ≈5 × 10^4^ g/mol), β-cyclodextrin (BCD, molar mass = 1134.98 g/mol), 2-hydroxypropyl-β-cyclodextrin (HPBCD, molar mass = 1460 g/mol), citric acid (molar mass = 210.14 g/mol), NaH_2_PO_2_•H_2_O (molar mass = 105.99 g/mol), methylene blue (MB, molar mass = 210.14 g/mol), pirfenidone (PFD, molas mass = 185.22 g/mol), rose Bengal (RB, molar mass = 1017.64 g/mol), glacial acetic acid 99.9% v/v and a 0.01M phosphate buffered saline solution (PBS; 0.138 M NaCl; 0.0027 M KCl; pH = 7.4 at 25°C) were obtained from Sigma-Aldrich (United States). Ethanol (EtOH, C_2_H_5_OH), acetic acid (CH_3_COOH), sodium hydroxide (NaOH), and hydrochloric acid (HCl) were purchased from Merck (Germany). All chemicals were used without further purification, and solutions were prepared using Milli-Q water with resistivity of 18.2 MΩcm (pH = 7.6, otherwise mentioned).

### 2.2 Solid-phase peptide synthesis

The antimicrobial peptide KR-12 (H-Lys-Arg-Ile-Val-Gln-Arg-Ile-Lys-Asp-Phe-Leu-Arg-NH_2_) was automatically synthesized with the microwave-assisted peptide synthesizer Liberty Blue (CEM, Matthews, NC, United States). All applied amino acids were Fmoc (fluorenylmethoxycarbonyl)-protected at the α-amino acid. In the case of arginine (Arg) the orthogonal protection group was 2,2,4,6,7-pentamethyldihydrobenzofuran-5-sulfonyl (Pbf), lysine and aspartic acid were *tert.*-butyl-protected (all amino acids from IRIS Biotech, Germany). For obtaining a C-terminal amide an unloaded Fmoc-Rink-Amide-resin was used (substitution grade 0.30 mmol/g, INTAVIS Peptide Service GmbH, Germany). The synthesis was performed in a 0.1 mmol scale on dimethylformamide (DMF) (IRIS Biotech, Germany) and the coupling was performed with DIC(Diisopropylcarbodiimid)/Oxyma (ethyl cyanohydroxyiminoacetate) (0.5 M/1.0 M, respectively). For deprotection of the Fmoc-group a 20% piperidine solution in DMF was used. The peptide was cleaved from the resin by a mixture of 95% TFA (trifluoroacetic acid)/2.5% water/2.5% triisopropylsilane. After the cleavage, the peptide was obtained by precipitation in ice-cold methyl tert-butylether. The precipitated peptide was centrifuged, and the supernatant was discarded. The remaining peptide was solubilized in UHQ water and freeze-dried in a lyophilizer (Martin Christ Alpha one to four, Germany). The obtained peptide was characterized by MALDI-ToFMS (Autoflex Speed, Bruker Daltonik, Germany) using α-CHCA (α-cyano-4-hydroxycinnamic acid) as a matrix. The mass of the desired KR12-peptide of 1,570.93 g/mol was confirmed and a purity ≥95% was targeted.

### 2.3 Silicone implant sample preparation

Samples were obtained from the surface of a smooth and textured implant. Each implant was cut around the contour using a scalpel, dividing them in half. Both parts were manually separated to remove the gel manually, and the residues were removed with ethanol. Subsequently, both surfaces were cut into 1 × 1 cm^2^ samples, rinsed with ethanol and dried.

### 2.4 Solutions preparation

Two CHI solutions (0.1% and 1% w/v) were prepared by dissolving the reagent in 570 µL of 100 mM glacial acetic acid and 90 mL of distilled water. The solution was stirred overnight on a magnetic stirrer (model MS-MP8, Witeg). Finally, the pH value was adjusted to 4 with a 0.1 M HCl and/or NaOH solution, and the volume of the solution was made up to 100 mL.

The cyclodextrin (BCD or HPBCD, Supplementary Figure S1) solution was prepared with 10 g of cyclodextrin, 3 g NaH_2_PO_2_•H_2_O as a catalyst, and 10 g of citric acid in 100 mL of distilled water. The solution was stirred for 1 h on a magnetic stirrer (model MS-MP8, Witeg) until the reagents were dissolved. MB solution was prepared in a concentration of 0.001 M at pH 7.0.

### 2.5 Pretreatment on samples

Implant samples were oxidized with oxygen plasma in a Harrick Plasma Cleaner model PDC-32G (United States). The equipment used a radio frequency power of 18W and was operated under 100 mL/min flow of O_2_ at a pressure of less than 0.2 mmHg for 15 min. Immediately after oxidation, the samples were immersed in the CHI solution (0.1 or 1% w/v) and placed on a horizontal shaker (model ZWY-103B, LABWIT) at 100 rpm for 15 min. Then, the samples were rinsed with double-distilled water at pH = 4 and dried at room temperature.

### 2.6 Functionalization of samples

After the pretreatment, samples were immersed in the cyclodextrin solution (BCD or HPBCD), stirring at 100 rpm for 15 min, followed by drying at 30°C. The cyclodextrin/citric acid polymerization was carried out in an oven (model ZFD-A540, Zhicheng) at 140°C for 30 min (Guzmán-Oyarzo et al., 2019). Finally, the samples were rinsed with double-distilled water and ethanol to remove unpolymerized residues and dried.

### 2.7 Pretreatment evaluation

The pretreatment performed on samples was evaluated by the capacity to absorb MB dye. Functionalized samples with and without the pretreatment step were immersed in the MB solution for 15 min. Then, samples were subjected to three consecutive rinses of 2, 1, and 1 min with distilled water and allowed to dry at room temperature. Afterward, the samples were immersed in a 50% v/v acetic acid solution to extract the MB dye. Finally, the absorbance values were obtained using a UV-Vis spectrophotometer (Evolution 220 model, Thermo Scientific) at 671 nm (Blanchemain et al., 2012).

### 2.8 Characterization techniques

The surface wettability of the samples was determined by a water contact angle measuring system (KSV CAM-101, Finland) on the static sessile drop mode. The volume of the water drop was 10 μL, and five measurements were carried out in different regions of each sample and an average value was reported.

Roughness measurements were carried out using a Dektak 150 stylus profilometer (Veeco, United States), applying a force of 1.0 mg and a scan speed of 17 μm/s.

Chemical analysis of the samples was performed by the Attenuated Total Reflectance Fourier- Transform Infrared Spectroscopy (ATR-FTIR). An FTIR spectrometer coupled with an ATR accessory using a zinc selenide crystal (CARY 630 FTIR Agilent Technologies, United States) was used between 4,000 and 600 cm^−1^ with a resolution of 1 cm^−1^ (NS = 4). The obtained spectra were mathematically processed by data smoothing and normalization.

The morphology of the samples was observed by optical microscopy (eclipse E200, Nikon) and by a variable pressure scanning electron microscope (VP-SEM, SU-3500 Hitachi, Japan) using an acceleration voltage of 5 kV. The size distribution of samples was presented as histograms; data were obtained from the SEM images that were processed using freely available ImageJ software.

### 2.9 Gravimetric analysis and hydrolytic degradation test

The degree of functionalization (mass%) was reported as the mass gain of the samples according to the following equation (Blanchemain et al., 2012):

$$Degree\;of\;functionalization\;\left(\%\right)=\left(\frac{M_{f}-M_{i}}{M_{i}}\right)\times 100\%$$ (1)

where $M_{i}$ y $M_{f}$ , corresponding to the mass of the sample before and after the treatment, respectively.

Hydrolytic degradation tests were conducted in PBS (pH 7.4) at 37°C using the gravimetric method. Mass loss measurements of the hydrogels in PBS was calculated with degradation time using the equation (Simmons and Kontopoulou, 2018):

$$Residual\;mass\;\left(\%\right)=\left(\frac{M_{i}-M_{f}}{M_{i}}\right)\times 100\%$$ (2)

where *M*_*i*_ is the initial mass of the sample and *M*_*f*_ is the final mass of the degraded sample extracted at each time period.

### 2.10 Drug release profile

Samples were loaded with PFD, RB, and KR-12 molecules (Supplementary Figure S2), using a concentrated solution of each compound and under stirring at 100 rpm: 1 mg/mL for PFD and RB for 12 h, and 0.5 mg/mL for KR-12 for 1 h. To obtain the drug release profiles, loaded samples were placed into vials filled with 5 mL of PBS at 37°C in a horizontal shaker (100 rpm) (NB-2005LN Biotek, Winooski, VT, United States). The supernatant solution was completely renewed at pre-determined time intervals, and the drug content in the withdrawn bulk fluid was analyzed by UV-spectrophotometry (Evolution 220 model, Thermo Scientific). Thus, PFD was detected at 310 nm (Parmar et al., 2014), RB at 545 nm (Hernández et al., 2016) and KR-12 at 208 nm (Yun et al., 2020). All experiments were conducted in triplicate, and non-functionalized samples were used as controls in the kinetic release experiments.

In order to determine the drug release mechanisms, the Korsmeyer-Peppas model model was firstly used for fitting. The Korsmeyer-Peppas semi-empirical model is given by (Wu et al., 2019):

MtM∞=kKPtn (3)

where *M*_*t*_ */M*_*∞*_ is the fractional drug release, *t* is the release time, *k*_*KP*_ is the Korsmeyer-Peppas release kinetic constant characteristic of the drug/system, and *n* is an exponent which characterizes the mechanism of release (Wu et al., 2019). Eq. 3 is used for generalized release data analysis, although it may only be used up to 60% of the released drug.

On the other hand, the monolithic solution model for a slab geometry was utilized to determine the diffusion coefficients of the functionalized samples (Siepmann et al., 2012):

MtM∞=4DtπL21/2 (4)

where *M*_*t*_ and *M*_*∞*_ denote the cumulative amounts of drug released at time *t* and at infinite time, respectively; *D* is the diffusion coefficient of the drug within the system, and *L* represents the total thickness of the film. Eq. 4 can be used for just up to 60% of the released drug. The fitting of the models was conducted with OriginLab software.

### 2.11 Cytotoxicity assays

Cytotoxicity was investigated using African green monkey kidney’s epithelial cells (Vero, ATCC-CCL-81) (Fernández Freire et al., 2009). Vero cells were obtained from National Institute for Quality Control in Health (INCQS), Oswaldo Cruz Foundation (Fiocruz), Rio de Janeiro, Brazil; AGS, ATCC-CRL-1739 cells were provided by Banco de Células do Rio de Janeiro, Brazil.

Vero cells were cultivated in RPMI medium (Cultilab, Campinas), supplemented with 10% (v/v) fetal bovine serum and incubated at 37 °C in an atmosphere with 5% of CO_2_. 1mL of the medium containing Vero cells was seeded in previously trypsinized 24-well culture plates at a cell concentration of 1x10^6^ with the breast implant with and without the peptide, and then incubated at 37°C at 5% of CO_2_ for 24 and 48 h. After the incubation time, the medium was removed and MTT (3-(4,5-dimethyl-2-thiazolyl)-2,5-diphenyl-2H-tetrazolium bromide) diluted with the RPMI medium was added; the samples were then incubated in 37°C at 5% of CO_2_ for 4 h. After this period, the plates were prepared for the MTT-tetrazolium method (Mosmann, 1983). The samples were shaken for 1 min on a plate shaker and the absorbance was measured at 570 nm in a microplate reader (ELx800, BioTek Instruments, Inc., Winooski, VT). Assays containing cells without samples were considered as positive control (+), which functioned as a benchmark for evaluating the influence of experiments with samples. In the case of assays with pristine samples, they were label as control.

### 2.12 Bacterial assays

The antibacterial activity assays were evaluated using *S. epidermidis* (ATCC 12228) and *Pseudomonas aeruginosa* (ATCC 27853) bacterial strain. These culture collections from the American Type Culture Collection (ATCC) were provided by the National Institute for Quality Control in Health (INCQS) – Oswaldo Cruz Foundation (Fiocruz, Rio de Janeiro, Brazil).

Firstly, the minimum inhibitory concentration (MIC) of the KR-12 peptide was determined by the agar dilution method as described by the guidelines from the Clinical Laboratory and Standards Institute (CLSI, 2015). Suspensions of the peptide in non-inoculated medium (negative control), and a stock solution of rifampicin at 5 μg/mL (positive control), were prepared in distilled water (20 μg/μL). The tested compounds were sequentially diluted with BHI (Brain and Heart Infusion KASVI) medium in a 96 multi-well plate for a final volume of 100 μL/well. An inoculum of 100 μL of bacteria in BHI suspension at 1.0 McFarland scale was added to each serial dilution in order to reach turbidimetric 0.5 McFarland (∼1.5 × 10^8^ CFU/mL; CFU = Colony-Forming Units) in a final volume of 200 μL/well. The final concentrations of the compounds ranged from 10,000–0.062 μg/μL. The multi-well plate was incubated for 18 h at 37°C in a humidity chamber. After incubation, 15 μL of resazurin at 0.02% in sterile aqueous solution were added to each well. The measurements were performed after 24 h of reincubation. All assays were performed in triplicate.

To determine the antibacterial effect of samples, the serial dilutions and plating technique were performed using aliquots of 1 × 10^6^ CFU/mL, which were inoculated in tryptic soy broth (TSB) medium containing the samples (1 × 1 cm^2^) in a safety cabinet (VLFS-12, Veco). The samples were incubated in triplicate for 24 h in a bacterial incubator (model AP-22, Phoenix) at 36 °C without culture media replacement. At the end of the 24 h period, the culture medium was removed to interrupt all growth. The samples were subsequently washed three times with deionized water to completely remove the constituents of the culture medium as well as non-attached cells and biofilms. The samples were then submitted to ultra-sonication in PBS to remove the attached cells and they were consecutively diluted with PBS at a proportion of 1:9 v/v. Aliquots of 0.2 mL of the obtained cell suspensions were then plated in triplicate onto solid agar medium using the spread plate method. After incubating for 24 h, the number of bacterial colonies was counted and the results, after multiplication by the dilution factor, were expressed as mean CFU per cm^2^. Bacterial cultures were performed in triplicate and survival rates were calculated by comparing CFU/cm^2^ of samples with immobilized peptides using the PEI substrate as control. Data were analyzed statistically by analysis of variance (ANOVA) with subsequent Tukey post-hoc test using Statistica 12.0 software; *p*-values of 0.05 or less were considered statistically significant. All assays were performed in triplicate.

## 3 Results and discussion

**FIGURE 1:**
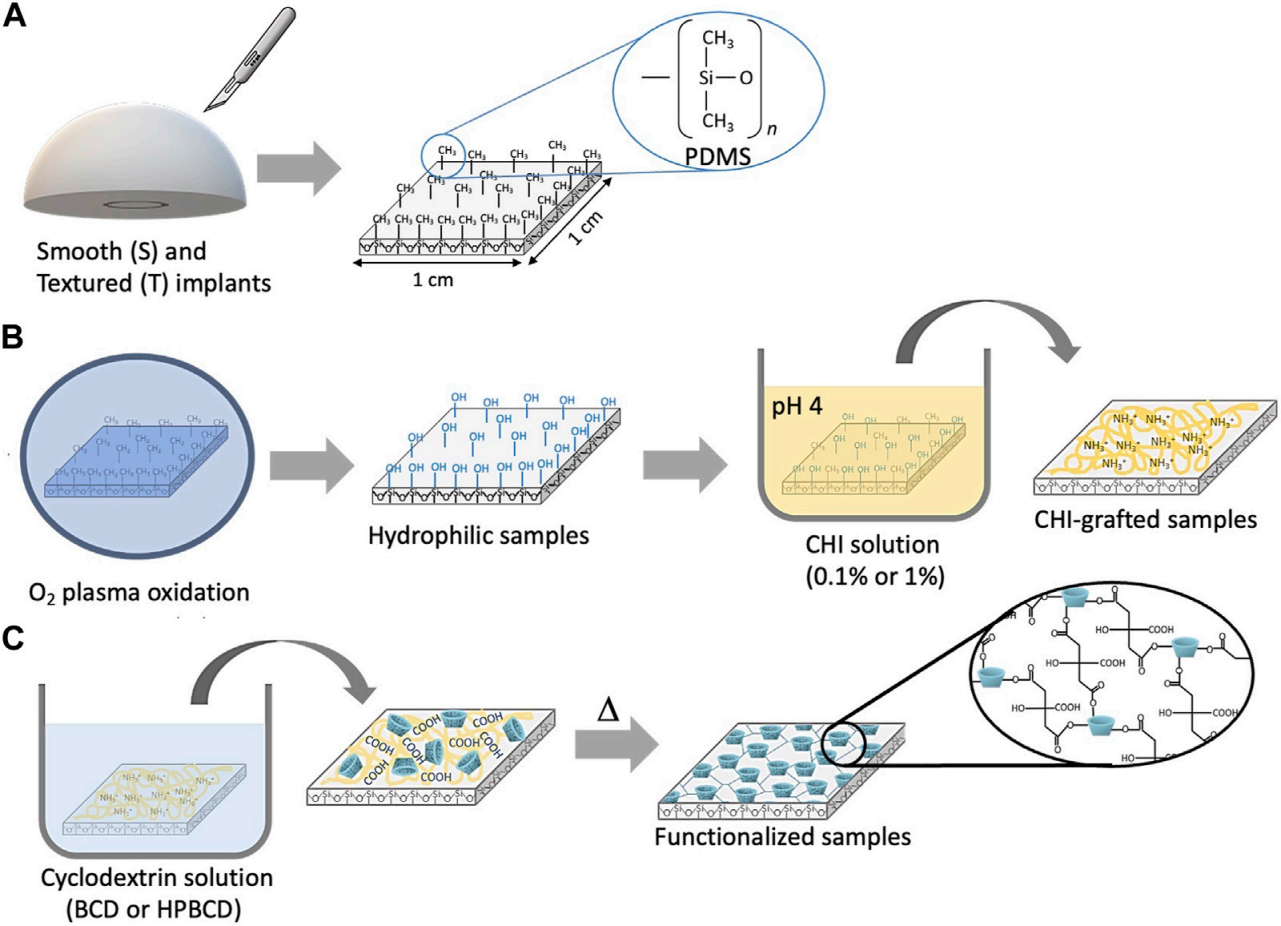
Schematic representation of the functionalization of samples: **(A)** Sampling, **(B)** pretreatment: plasma oxidation and chitosan grafting, and **(C)** cyclodextrin *in-situ* polymerization. The scheme is for reference only; the chemical changes are idealized.

In this study, samples were obtained from the surface of smooth (S) and textured (T) commercial breast implants (Figure 1A). As polydimethylsiloxane (PDMS) is the basis for both breast implant silicone shell (Daniels, 2012), the surface of these types of implants is hydrophobic. Therefore, before functionalizing the samples, a surface pretreatment of oxygen plasma oxidation with a subsequent chitosan grafting (0.1% or 1% w/v) was carried out (Figure 1B). After that, *in-situ* polymerization (BCD or HPBCD) in the presence of citric acid was performed (Figure 1C). The oxidation step is intended to integrate silanol groups on the implant surface. Consequently, the attachment of CHI would result in the formation of a coating through hydrogen bond interactions between hydroxyl groups and the partially amidated CHI, as the solution was prepared in an acidic medium (Chessa et al., 2016). Likewise, CHI generate an electrostatic bond between the oxidized surface and the copolymers (BCD or HPBCD with citric acid), which are also partially negatively charged due to their unreacted carboxylic groups of the copolymerized citric acid (Hernández-Montelongo et al., 2018): oxidized surface (−)/chitosan grafting (+)/cyclodextrin polymer (−).

**FIGURE 2:**
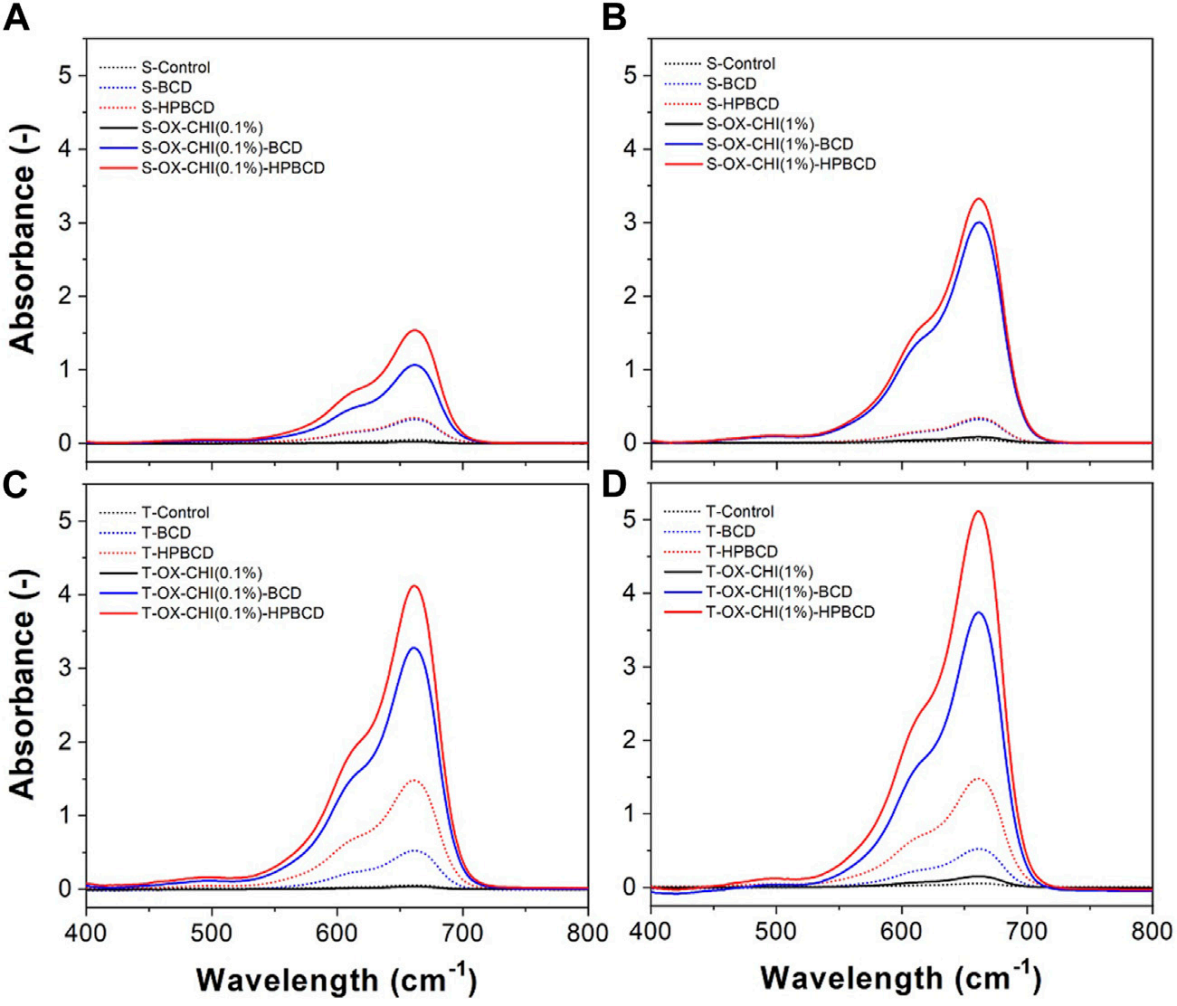
Methylene blue absorbance spectra obtained from the sequence of the treatments performed on the samples: **(A)** smooth with CHI 0.1%, **(B)** smooth with CHI 1%, **(C)** rough with CHI 0.1%, and **(D)** rough with CHI 1%.

The MB absorbing capacity was used to evaluate the pretreatment step of samples. This dye is positively charged and can be used to detect cyclodextrin/citric acid polymers, which are negatively charged (Hernández-Montelongo et al., 2018). Figure 2 shows representative MB absorbance spectra of samples with or without pretreatment and functionalization: plasma oxidation (OX) + CHI grafting (0.1% or 1%), followed by the cyclodextrin polymerization (BCD or HPBCD) on the implants surface (S or T). The control samples did not present an MB signal, confirming the absence of polymer. In the case of samples directly polymerized with no pretreatment presented some MB absorbance peaks: 0.35 for both S-BCD and S-HPBCD, 0.54 for T-BCD, and 1.5 for T-HPBCD. On the other hand, all samples with the pretreatment but without the polymerization process presented practically undetectable absorbance peaks. Nevertheless, a considerable MB absorbance increase was observed across in all the samples subjected to the pretreatment step and polymerization process. It is important to highlight that in all cases, samples with HPBCD presented higher MB absorbance values than BCD. Although it is known that BCD forms a 1:1 complex with MB, while HPBCD has a 2:1 inclusion complex (Kacem et al., 2013; Martin et al., 2013), which means twice HPBCD molecules are needed as BCD to encapsulate the same amount of MB. The higher MB absorbed in HPBCD samples is explained by the higher degree of polymerization that the HPBCD molecule generates as compared to native BCD. The hydroxypropyl groups of HPBCD improve the reactivity of the–OH groups present in the cyclodextrin, facilitating the esterification reactions with the cross-linking agent (Blanchemain et al., 2007; El Ghoul et al., 2010).

Figure 2 also shows the absorbance spectra of the samples treated with different CHI solution. Smooth samples treated with CHI 1% showed higher MB absorbance than smooth samples with CHI 0.1%: 1.1 and 1.5 maximal absorbance values for S-OX-CHI(0.1%)-BCD and S-OX-CHI(0.1%)-HPBCD, respectively. These values increased up to 3 and 3.3 for S-OX-CHI(1%)-BCD and S-OX-CHI(1%)-HPBCD, respectively, as shown in Figures 2A, B. In the case of textured samples, the increase of CHI concentration did not reflect the same proportional increase in MB absorbance as in smooth samples: 3.3 and 4.1 absorbance values for T-OX-CHI(0.1%)-BCD and T-OX-CHI(0.1%)-HPBCD, respectively, increased up to 3.7 and 5.1 for T-OX-CHI(1%)-BCD and T-OX-CHI(1%)-HPBCD, respectively, as can be seen in Figures 2C, D. These results suggest that the roughness of textured samples plays a key role in the polymerization. The protrusion in the surface could work as anchoring of the cyclodextrin polymers (Hernández-Montelongo et al., 2014). A higher amount of polymer was forming the films in the textured surfaces, even with CHI 0.1%. Due to the higher amounts of obtained using the concentration of CHI 1%, this was chosen as a condition for the pretreatment step.

**FIGURE 3:**
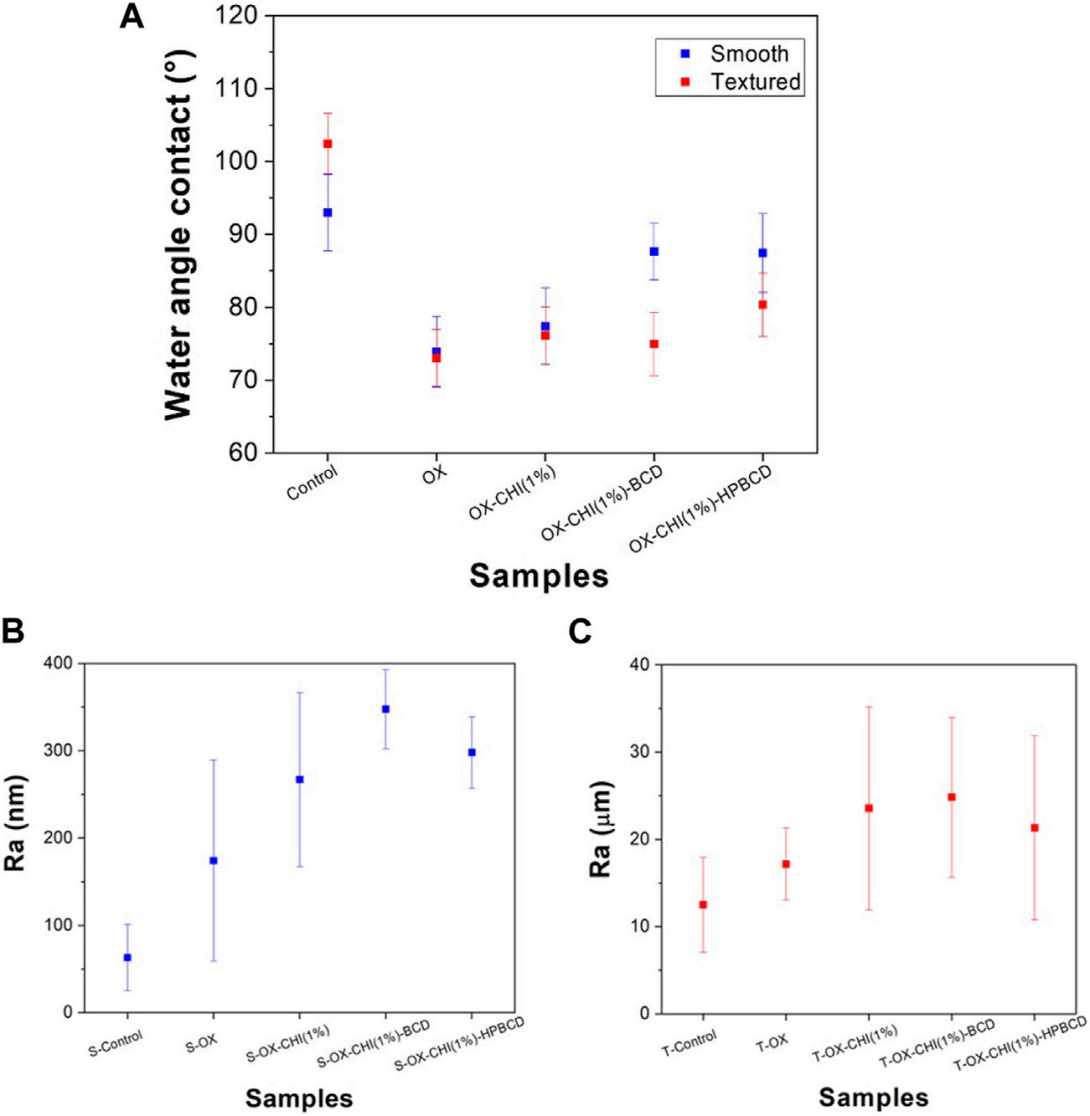
**(A)** Water contact angle for smooth and textured samples according to the sequence of chemical treatments performed. Surface roughness after each chemical treatment carried out on **(B)** smooth samples and **(C)** textured samples.

To detect changes on the surface after the chemical treatments, the wettability and roughness of the samples were measured (Figure 3). Figure 3A shows the water contact angle of the smooth and textured samples. Both control samples, smooth and textured, are considered hydrophobic (angles of 93° ± 5° and 121° ± 10°, respectively), due to the–Si-CH3 groups present on the surface of the PDMS surface (Mor et al., 1990). After the plasma oxidation, the contact angle decreased to 74° ± 5° and 72° ± 8° on the smooth and textured samples, respectively. These results indicate a hydrophilic behavior due to the formation of silanol groups (–Si-OH) on the surface (Peterson et al., 2005). Thus, this allowed the CHI to bind to the activated PDMS surface by hydrogen bridges. After the CHI grafting, samples remained hydrophilic, and the contact angle was practically the same for both types of surfaces (77° ± 5° for the smooth and 77° ± 10° for the textured).

In the case of polymerized samples, the contact angle increased to 88° ± 4° and 87° ± 5° on the smooth surface, BCD and HPBCD, respectively; while in the textured samples, the angles were 73° ± 12° for BCD and 84° ± 7° for HPBCD. The hydrophilicity of polymerized samples comes from the cyclodextrin functional groups interaction (COOH, OH) with the water and the residual carboxylic acid groups present in the cross-linking agent (citric acid) (Blanchemain et al., 2007; El Ghoul et al., 2010).

Figures 3B, C show the smooth and textured average roughness values, respectively. The smooth samples showed values in a range of hundreds of nanometers, and the textured in the scale of tens of microns. After each chemical treatment step the roughness increased; obtaining similar tendency in both surface types. The smooth surface sample increased from 63 ± 38 nm (control) to 348 ± 45 nm and 298 ± 40 nm for the samples polymerized with BCD and HPBCD, respectively. In the case of the textured samples, the roughness increased from 12.5 ± 5 µm (control) to 25 ± 9 μm and 21 ± 10 µm for the samples polymerized with BCD and HPBCD, respectively.

**FIGURE 4:**
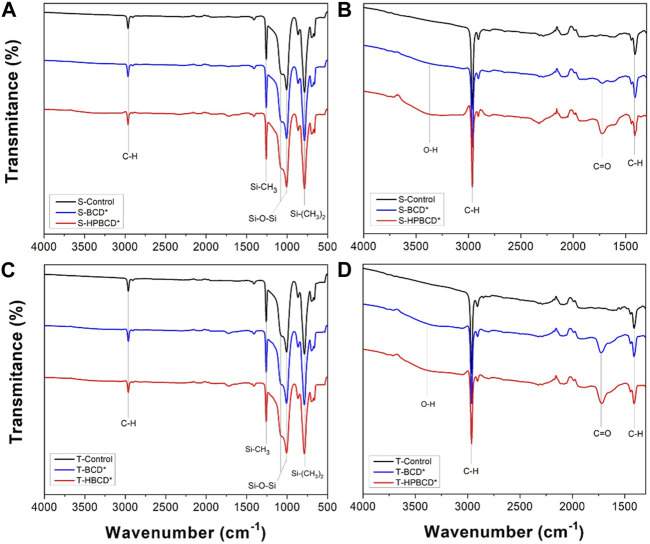
FTIR spectra of samples, with smooth (**A, B** (magnification from **A**)) and textured (**C, D** (magnification from **C**)) surfaces, functionalized with BCD and HPBCD.

In order to make the reading easier, the functionalized samples were relabeled as S-BCD*, S-HPBCD*, T-BCD* and T-HPBCD* to substitute S-OX-CHI(1%)-BCD, S-OX-CHI(1%)-HPBCD, T-OX-CHI(1%)-BCD, and T-OX-CHI(1%)-HPBCD, respectively. The chemical analysis was performed by ATR-FTIR to directly identify the surface modification (Figure 4). The bending vibrations of the fingerprint functional groups of PDMS were detected. The Si-(CH_3_)_2_ (786 cm^–1^), symmetric bending of Si-CH_3_ (1263 cm^–1^), stretching of CH (2,960 cm^–1^) from CH_3_, Si-O-Si stretching at 1065 cm^–1^ and 1007 cm^–1^ were observed in the spectra of both controls and functionalized samples (Bodas and Khan-Malek, 2006). Figures 4B, D show the spectra of the cyclodextrin functionalized samples. Characteristic functional groups of cyclodextrin polymers were detected: O-H stretching of the hydroxyl and carboxyl groups (3,435 cm^–1^) and the C=O stretching (1736 cm^–1^) of ester groups resulted from the esterification and the residual carboxylic acids of the cross-linking agent (Zhao et al., 2009). It is essential to mention that these two signals were not detected in both substrate controls. In the case of the smooth samples (Figure 4B), the intensity of O-H and C=O functional groups was higher for S-HPBCD* than S-BCD*, which indicates that there were more esterification reactions using HPBCD molecule than BCD. In the textured samples (Figure 4D), T-BCD* and T-HPBCD* showed both O-H and C=O groups with similar intensity, confirming the key role of roughness for the *in-situ* polymerization.

**FIGURE 5:**
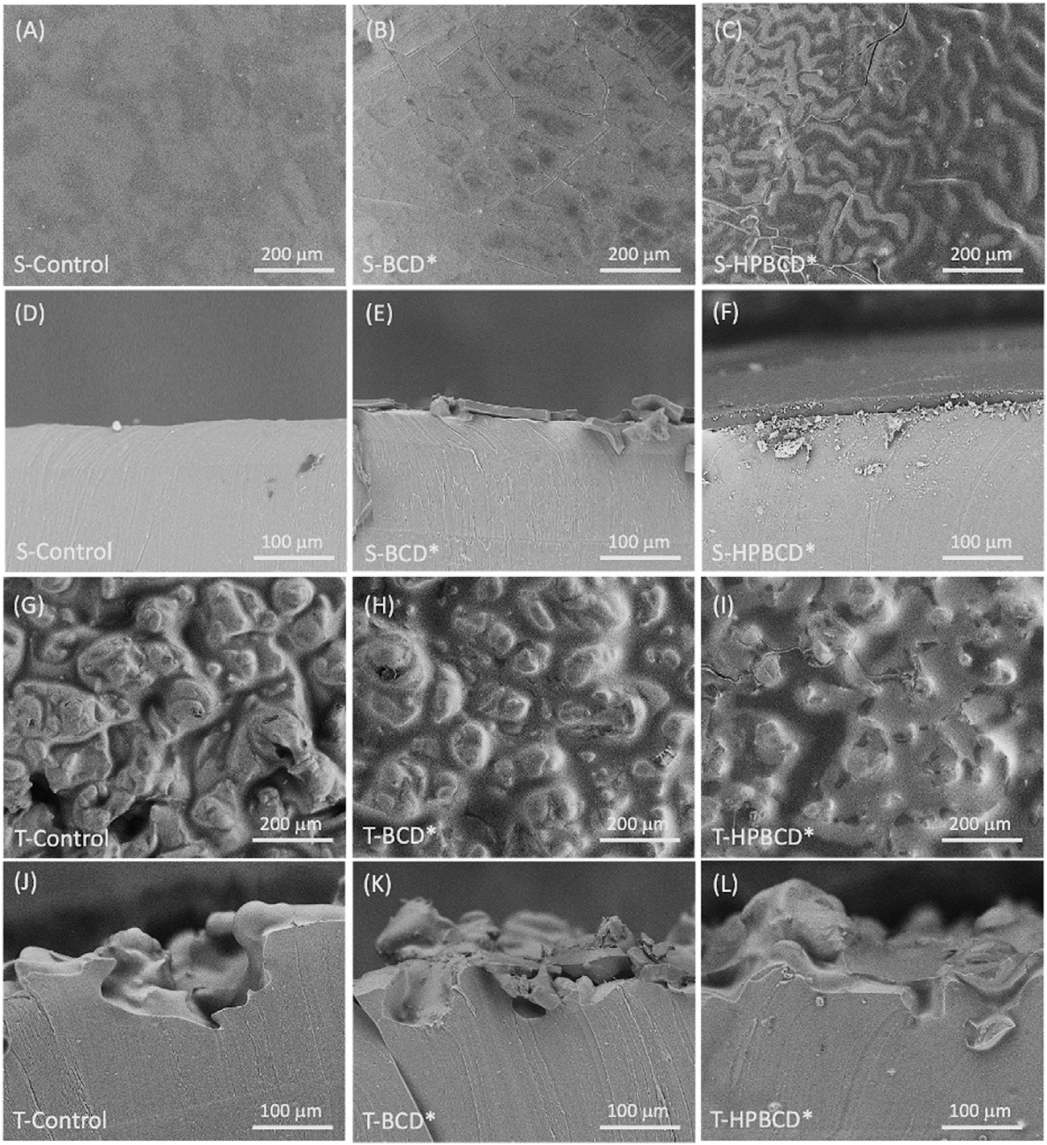
SEM images of samples: surface view of S-Control **(A)**, S-BCD* **(B)**, S-HPBCD* **(C)**; cross-sectional view of S-Control **(D)**, S-BCD* **(E)**, S-HPBCD* **(F)**; surface view of T-Control **(G)**, T-BCD* **(H)**, T-HPBCD* **(I)**; cross-sectional view of T-Control **(J)**, T-BCD* **(K)**, T-HPBCD* **(L)**.

Figure 5 shows the top view and cross-section SEM images of the control and functionalized samples. Regarding the smooth implants, the top view of S-Control exhibits a completely flat surface (Figure 5A), while the functionalized samples exhibited textures and folds attributed to the biopolymers, Figure 5B for S-BCD* and Figure 5C for S-HPBCD*. The layer thickness, obtained by the cross-section, was 14 ± 3 μm and 20 ± 3 µm for S-BCD* and S-HPBCD*, respectively. The images of textured samples showed a rough surface with structures of approximately 200 μm; valleys with a deep down to 100 µm (Figures 5G, J for the T-Control). In the case of functionalized samples, the valleys were filled, exposing partially the surface peaks. T-HPBCD* (Figure 5 I and 5 L) showed a better coverage of the substrate surface than T-BCD* (Figure 5 H and 5 K), suggesting a higher polymerization degree using HPBCD than BCD.

Table 1 presents the degree of functionalization and degradation parameters (Equations 1 and 2). Results indicate that the degree of functionalization on textured samples was higher than smooth ones, confirming the key role of the initial control surface roughness. Additionally, the functionalization degree was higher using HPBCD than BCD on both substrates, in agreement with previous characterization analyses.

**TABLE 1:** Degree of functionalization and parameters obtained from hydrolytic degradation tests.

Sample	Degree of functionalization (mass%)	Degradation time (days)	Degradation (mass%)	Degradation (%)	Degradation rate (day^-1^)
S-Control	–	–	–	–	–
S-BCD*	1.4 ± 0.5	∼7	0.9	64	0.0011
S-HPBCD*	2.5 ± 0.5	∼7	1.9	76	0.0025
T-Control	–	–	–	–	–
T-BCD*	3.7 ± 0.5	∼11	1.7	46	0.0012
T-HPBCD*	5.3 ± 0.5	∼11	2.8	53	0.0021

**FIGURE 6:**
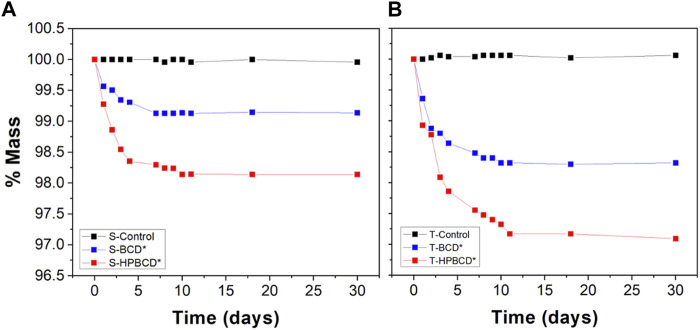
Residual mass as a function of hydrolysis time in PBS medium at pH 7.4°C and 37°C: **(A)** Smooth and **(B)** textured samples.

The hydrolytic degradation results are shown in Figure 6. The polymeric films on textured samples were more stable than smooth ones, around 11 and 7 days, respectively. Degradation rates were higher in samples functionalized with HPBCD than BCD. This is because HPBCD samples presented a higher amount of polymer forming the layers, then, the polymer was degraded faster. Remarkably, for all cases, the polymer was not totally degraded after a month in the hydrolytic assays fluid for all the cases.

**FIGURE 7:**
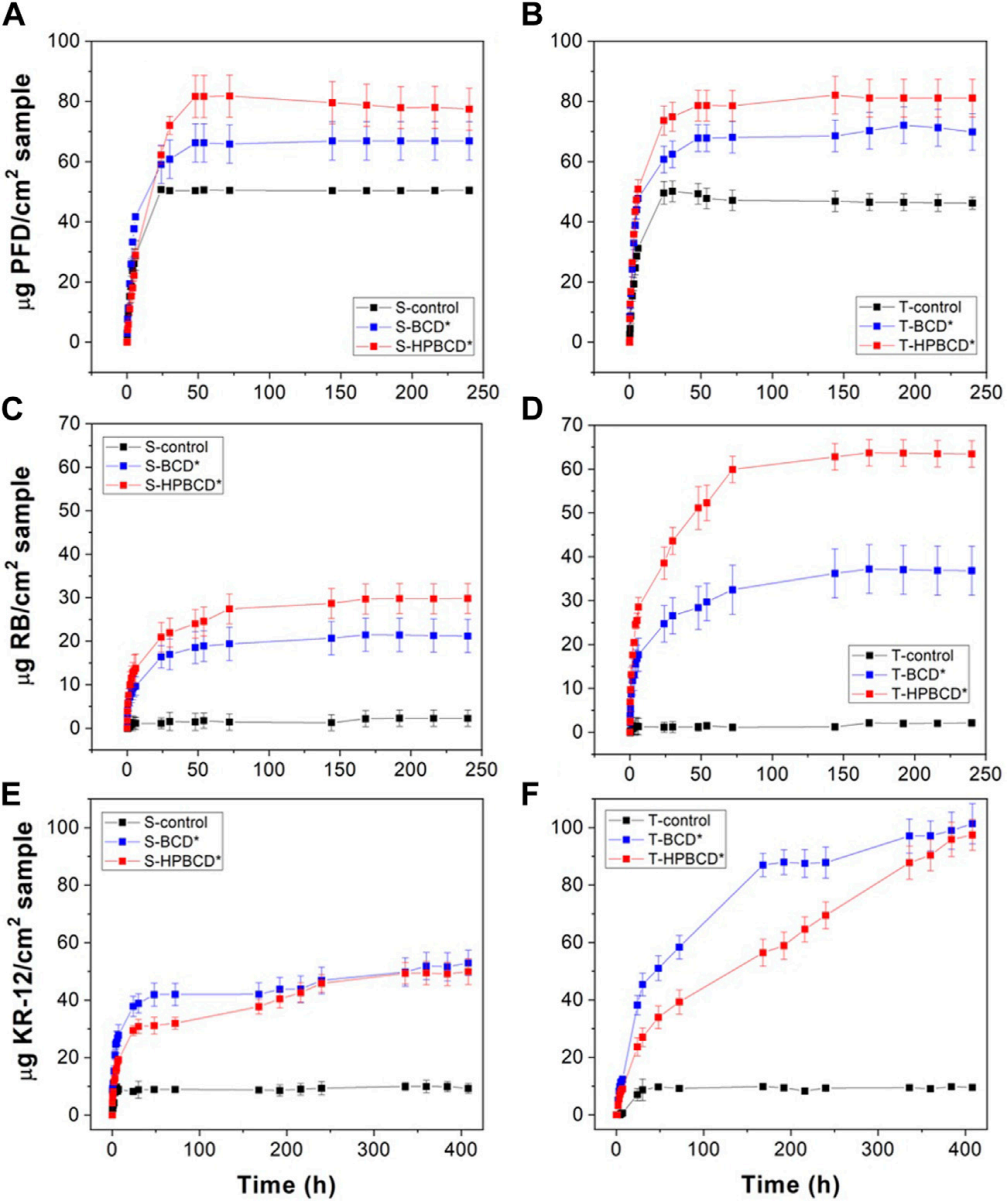
Drug release profiles of: PFD release from smooth **(A)** and textured **(B)** samples, RB release from smooth **(C)** and textured **(D)** samples, and, KR-12 release from smooth **(E)** and textured **(F)** samples.

To evaluate the drug delivery functionality of samples, they were tested with PFD, RB, and KR-12 in PBS batches at 37°C under stirring. Controls and functionalized samples were loaded with highly concentrated solutions of PFD, RB, and KR-12. The obtained drug release profiles are shown in Figure 7. The maximum released amount of each drug, reached in the equilibrium time, is reported in Table 2. Results show that both pristine samples (S-Control and T-Control) were able to release considerable amounts of PFD, demonstrating the chemical compatibility between the chemical structure of PFD and silicone substrates (Wu et al., 2021). In the case of functionalized samples, those polymerized with HPBCD presented higher amounts of released PFD than samples polymerized with BCD. Independently of the type of substrate, BCD and HPBCD samples released around 38% and 62% higher than controls, respectively. In the RB release profiles, both controls released negligible amounts of RB, and in the case of polymerized samples, the released mass depended on the type of cyclodextrin and substrate. These results mean that functionalized samples released in the order of thousands of times higher mass of RB than controls. Moreover, it is important to highlight that, for both molecules, PFD and RB, functionalized samples reached higher equilibrium times than controls. In the case of the antimicrobial peptide release profiles, the controls released same amounts of KR-12. Functionalized smooth samples released similar values between them, and functionalized textured samples reached also similar values. In this experiment series, the equilibrium time was not reached even at 400 h. This could be due to the high molecular weight of the KR-12 peptide (1,570.95 g/mol) in comparison with RB (973.67 g/mol) and PFD (185.22 g/mol) since high molecular weight molecules present, except for specific surrounding environments, lower diffusion coefficients (Bogdan et al., 2011).

**TABLE 2:** Maximum amount of each drug released from samples in PBS at 37°C, reached in the equilibrium time.

Sample	PFD (μg/cm^2^) and equilibrium time	RB (μg/cm^2^) and equilibrium time	KR-12 (μg/cm^2^) and equilibrium time
S-Control	51 ± 1 in 24 h	2 ± 2 in 24 h	10 ± 2 in 24 h
S-BCD*	67 ± 7 in 48 h	21 ± 4 in 72 h	53 ± 4 in 400 h†
S-HPBCD*	82 ± 7 in 48 h	30 ± 3 in 72 h	50 ± 4 in 400 h†
T-Control	50 ± 3 in 24 h	2 ± 1 in 72 h	10 ± 1 in 24 h
T-BCD*	72 ± 6 in 48 h	37 ± 6 in 144 h	101 ± 7 in 400 h†
T-HPBCD*	82 ± 6 in 48 h	64 ± 3 in 144 h	97 ± 6 in 400 h†

^a^ The equilibrium was not reached.

To attain a deeper perception of the mechanisms that govern the release of PFD, RB, and KR-12 from the functionalized samples, release profiles were fitted to the Korsmeyer-Peppas model (Wu et al., 2021). The diffusion coefficients were obtained using the monolithic solution model (Siepmann et al., 2012). Both models were fitted to the normalized experimental data (Supplementary Figure S3). The obtained kinetic parameters from them are presented in Table 3. According to the *r* ^*2*^_*adj*_ obtained in all samples, both models fit well to the experimental data. In the case of the Korsmeyer-Peppas model, the kinetic constant *k*_*KP*_, which represents the release rate of a molecule (Bruschi and Bruschi, 2015), presented similar values among all samples, suggesting a similar kinetic rate between them. On the other hand, the release exponent n = 0.5, or n ≤ 0.5 corresponds to a Fickian diffusion release during which the solvent penetration is the rate-limiting step; 0.5 < n < 1.0 is related to non-Fickian release, this means that drug release followed both diffusion and erosion mechanisms; and n = 1 corresponds to zero order release, where drug release is independent of time applications (Hernández-Montelongo et al., 2014). According to this, the release of PFD from the four types of samples (S-BCD*, S-HPBCD*, T-BCD*, and T-HPBCD*) was controlled by both diffusion and erosion mechanisms. In the case of the other molecules, RB and KR-12 were mainly controlled by diffusion. These results can be explained by the molecular weight and size of molecules. In a determined volume of the cyclodextrin polymers, it is expected to find more smaller molecules (PFD) than bigger ones (RB and KR-12). Then, the erosion of the polymer releases more small molecules than big ones. That is why erosion was significant in the PFD experiments and not in the RB and KR-12 assays.

**TABLE 3:** The release kinetics parameters of PFD, RB, and KR-12 from functionalized samples in PBS at 37°C.

Molecule	Sample	Korsmeyer-Peppas $\frac{M_{t}}{M_{\infty }}=k_{KP}t^{n}$			Monolithic solution MtM∞=DtπL212	
		$k_{KP}\;\left(h^{-n}\right)$	n	$r_{adj}^{2}$	$D\;\left(\mu m^{2}/h\right)$	$r_{adj}^{2}$
PFD (185.22 g/mol)	S-BCD*	0.1732	0.7300	0.9959	2.1307	0.9536
	S-HPBCD*	0.0910	0.6726	0.9906	1.4702	0.9395
	T-BCD*	0.2293	0.6113	0.9917	132.6139	0.9784
	T-HPBCD*	0.2162	0.6083	0.9930	121.9176	0.9804
RB (973.67 g/mol)	S-BCD*	0.2255	0.3446	0.9952	1.8714	0.9184
	S-HPBCD*	0.2530	0.3321	0.9865	3.47503	0.9597
	T-BCD*	0.2370	0.3451	0.9712	82.4132	0.9914
	T-HPBCD*	0.2172	0.3430	0.9723	46.4713	0.8608
KR-12 (1,570.95 g/mol)	S-BCD*	0.2691	0.3211	0.9659	1.8347	0.9450
	S-HPBCD*	0.1811	0.3671	0.9950	1.2890	0.9299
	T-BCD*	0.0580	0.5355	0.9729	8.9724	0.9703
	T-HPBCD*	0.0423	0.5136	0.9849	3.9941	0.9828

**FIGURE 8:**
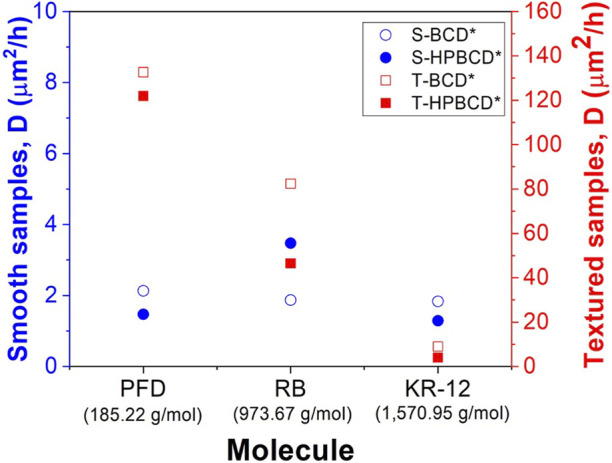
Diffusion coefficients of the different systems for the released molecules from the functionalized samples.

Regarding the monolithic solution model, the obtained diffusion coefficients (*D*) from Table 1 are plotted in Figure 8 for an easier data analysis and correlation. The results indicate that the diffusivity of molecules from the smooth samples, independently of the cyclodextrin polymer, showed similar values (from 1.3 to 3.5 μm^2^/h). However, in the case of textured samples, the diffusivity depended on the molecular weight (Bogdan et al., 2011). Therefore, lower molecular weights presented higher diffusivity and *vice versa*. The surface area of samples can explain this phenomenon. The high roughness of textured samples, 100 times higher than smooth samples, also represents a higher surface area for textured samples than smooth ones; then, the mass diffusion should be favored. That is why the PFD molecule showed the highest diffusion coefficient in the textured samples (132.6 and 121.9 μm^2^/h for T-BCD* and T-HPBCD*, respectively); RB presented the medium values (82.4 and 46.4 μm^2^/h for T-BCD* and T-HPBCD*, respectively), and KR-12 exhibited the lowest values (8.9 and 3.9 μm^2^/h for T-BCD* and T-HPBCD*, respectively). We suggest that in the case of the smooth samples, the diffusion coefficients were independent of the molecular weight because the polymer on the top surface substrate had less steric conformation restrictions for movement and diffusion. In all cases of smooth samples, *D* values were in the range of 1.3–3.5 μm^2^/h.

**FIGURE 9:**
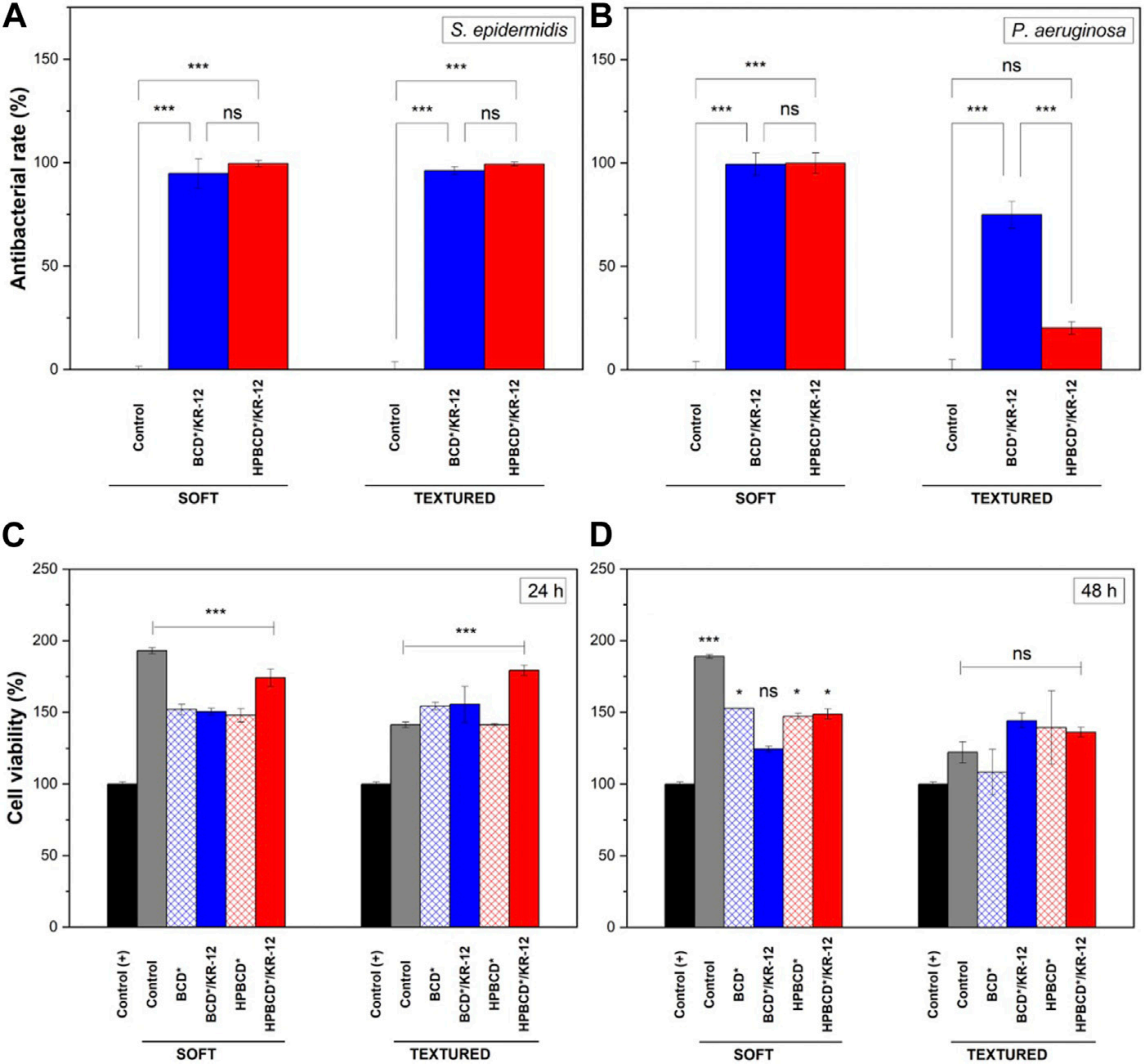
Antibacterial tests of implants loaded with KR-12: **(A)** *Staphylococcus epidermidis*, and **(B)** *Pseudomonas aeruginosa*. Cytotoxicity assay on vero cells after: **(C)** 24 and **(D)** 48 h. Results represent mean ± SD of three measurements, statistically interpreted by analysis of variance (ANOVA) with subsequent Tukey post-hoc test. Significant differences are presented as * is *p*-value ≤0.05 and *** is *p*-value ≤0.001, both vs. positive control (+). ns means not statistically significant.

After physicochemical characterization and drug release studies, the antibacterial effect of the KR-12 was evaluated. It has been reported that KR-12 possess antimicrobial and antibiofilm properties against *S. epidermidis* (Gram-positive) and *P. aeruginosa* (Gram-negative) bacteria (M et al., 2019; Ajish et al., 2022). In that sense, first, the minimum inhibitory concentration (MIC) of peptide in solution was obtained for both bacteria, which are two of the most virulent strains in breast implant infections (Chessa et al., 2016; Mesa et al., 2021). The results were 100 μg/mL for *S*. *epidermidis* and 200 μg/mL for *P*. *aeruginosa*, respectively. The enhanced resistance of the *P. aeruginosa* against KR-12 can be explained by the outer cell membrane, typical of Gram-negative bacteria. Afterwards, the antibacterial effect of the implants loaded with KR-12 was tested (Figure 9). In the case of *S*. *epidermidis* strain, all funcionalized samples, with the two types of implants and the two polymers, showed almost 100% of antibacterial rate in comparision with the unfunctionalized samples. In the case of the *P*. *aeruginosa* strain, the antibacterial effect was mainly observed in the functionalized soft implants (S-BCD* and S-HPBCD*), with also 100% of antibacterial rate in comparision with the control samples. However, textured samples presented a low antibacterial rate for *P*. *aeruginosa* bacteria: 75% for T-BCD* and 20% for T-HPBCD*. This can be explained due to their low diffusivity values (Figure 8); for these samples, MIC values of KR-12 for *P*. *aeruginosa* were not totally released at 24 h of bacterial culture.

Concerning the cytotoxicity of the implants against mammalian cells, due to the viability of Vero cells after 24 and 48 h of treatments (Figures 9C, D, respectively) is possible to see that the implants showed no cytotoxic profile, when they were compared with the positive control (+). In fact, during the first 24 h, samples increased the cell density up to 1.5–2 times. This phenomenon can be explained by the fact that cells tend to preferentially grow on rough substrates (Gentile et al., 2010; Ferrari et al., 2019). In the case of the control (+), where no motifs, neither geometric nor biochemical for cell adhesion are present, cell viability values for both the control and the samples were notably higher. In the case of 48 h, samples started to exhibit a reduction in the cell density, but the values were higher than for the positive control (+). The same behavior was also observed in samples loaded with KR-12 peptide.

## 4 Conclusion

The functionalization of breast implants through cyclodextrin *in-situ* polymerization was obtained by an oxidation pretreatment step with O_2_ plasma and CHI 1% w/v grafting. CHI facilitated an electrostatic bond between the oxidized surface and the BCD or HPBCD polymers. Among the samples, the textured sample polymerized with HPBCD exhibited the highest polymerization yield and stability. The polymer thickness on the top surface varied with the substrates: smooth samples had a 14–20 µm thickness, while textured samples had a thickness of approximately 100 µm. The diffusivity values (*D*) for the smooth samples ranged from 1.3 to 3.5 μm^2^/h. For the textured samples, the diffusivity depended on the molecular weight: PFD (185.22 g/mol) showed the highest *D* values (121.9–132.6 μm^2^/h), RB (1017.64 g/mol) exhibited medium values (46.4–82.4 μm^2^/h), and KR-12 (1,570.95 g/mol) displayed the lowest values (3.9–8.9 μm^2^/h). Notably, KR-12 demonstrated successful antibacterial activity against *S. epidermidis* with an antibacterial rate close to 100%. The antibacterial rate against *P. aeruginosa* bacteria varied, showing dependence on the diffusivity values. Furthermore, the functionalized samples were tested with epithelial cells and confirmed to be non-cytotoxic. Although the performed *in vitro* assays have confirmed the improved properties of functionalized samples compared to unprotected implants, it is advisable to conduct further research involving biological molecular and *in vivo* experiments to gain a deeper understanding of the therapeutic effects of the controlled release molecules in this drug delivery system.

## Data Availability

The raw data supporting the conclusion of this article will be made available by the authors, without undue reservation.
