# Supplementary material for: Functionalization of breast implants by cyclodextrin in-situ polymerization: a local drug delivery system for augmentation mammaplasty
Source: Front Bioeng Biotechnol. 2023 Sep 19;11:1254299. doi: 10.3389/fbioe.2023.1254299 (PMC10557261; doi:10.3389/fbioe.2023.1254299)
Supplement: Supplementary file 1 [file DataSheet1.PDF]

# Functionalization of breast implants by cyclodextrin in-situ polymerization: a local drug delivery system for mammoplasty

Karen Escobar, Ignacio Carrera, Nelson Naveas, Ruth Pulido, Miguel Manso, Joao Paulo de Oliveira Guarnieri, Marcelo Lancellotti, Monica A. Cotta, Yendry Regina Corrales-Ureña, Klaus Rischka, Jacobo Hernandez-Montelongo

## Supplementary Information

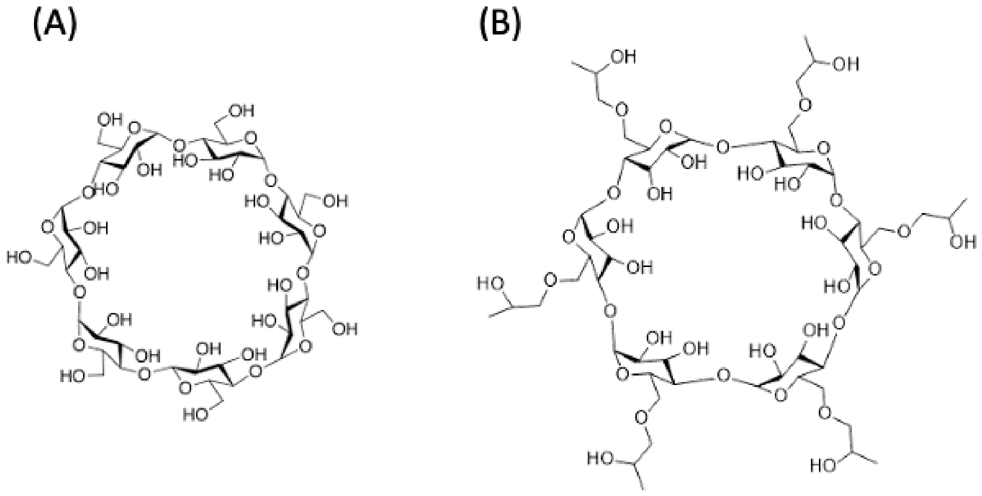


**Figure S1.** Chemical structure of: (A) β-cyclodextrin, and (B) 2-hydroxypropyl-β-cyclodextrin.

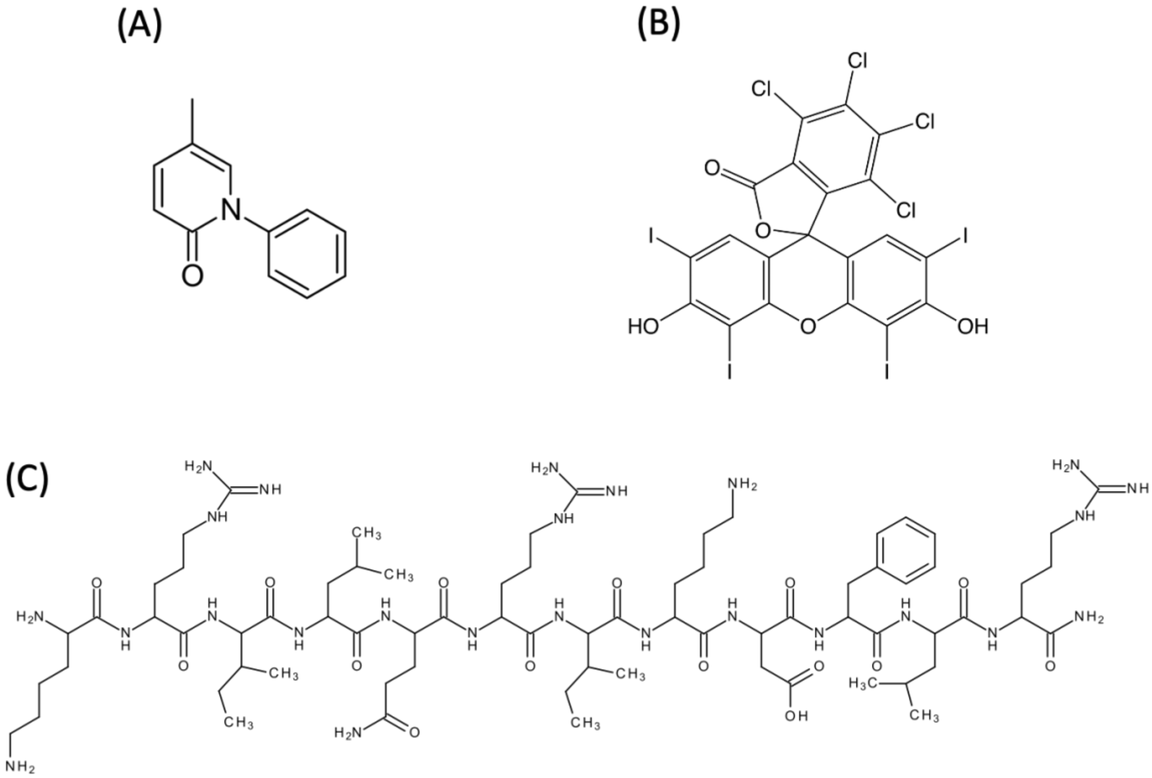


**Figure S2.** Chemical structure of: (A) pirfenidone, (B) rose Bengal, and (C) KR-12 antimicrobial peptide.

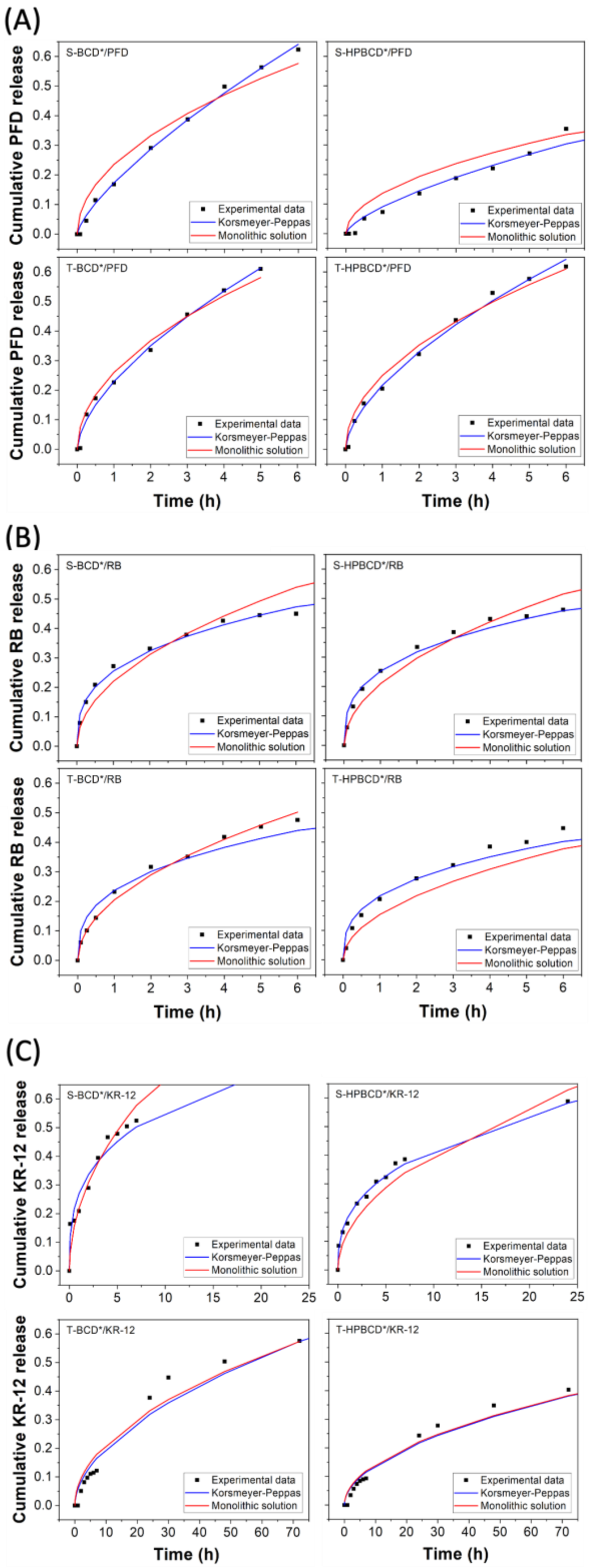


**Figure S3.** Normalized release profiles and fitted to the Korsmeyer-Peppas and monolithic solution models for the cases of: (A) pirfenidone, (B) rose Bengal, and (C) KR-12 antimicrobial peptide. Mathematical models were performed for slab geometry and up to 60% of the cumulative drug release.
